# Fungus-mediated Extracellular Biosynthesis and Characterization of Zirconium Nanoparticles Using Standard *Penicillium* Species and Their Preliminary Bactericidal Potential: A Novel Biological Approach to Nanoparticle Synthesis

**DOI:** 10.22037/ijpr.2019.112382.13722

**Published:** 2019

**Authors:** Ahmad Reza Golnaraghi Ghomi, Mohammad Mohammadi-Khanaposhti, Hossein Vahidi, Farzad Kobarfard, Mahdieh Ameri Shah Reza, Hamed Barabadi

**Affiliations:** a *Fouman Faculty of Engineering, College of Engineering, University of Tehran, Fouman, Iran.*; b *Department of Pharmaceutical Biotechnology, School of Pharmacy, Shahid Beheshti University of Medical Sciences, Tehran, Iran. *; c *Department of Medicinal Chemistry, School of Pharmacy, Shahid Beheshti University of Medical Sciences, Tehran, Iran.*

**Keywords:** Nanobiotechnology, Green synthesis, Penicillium family, Zirconium nanoparticles, Antibacterial activity

## Abstract

Biological synthesis of nanoparticles (NPs) has gained extensive attention during recent years by using various biological resources such as plant extracts and microorganisms as reducing and stabilizing agents. The objective of the present study was to biosynthesize zirconium NPs using *Penicillium* species as a reliable and eco-friendly protocol for the first time. The synthesized NPs were characterized using Scanning Electron Microscope (SEM), Atomic Force Microscope (AFM), Dynamic Light Scattering (DLS), Energy Dispersive X-ray (EDX), and Fourier Transform Infrared (FT-IR). The results showed that three *Penicillium* species were able to synthesize zirconium NPs extracellularly with spherical morphology below 100 nm. Moreover, the preliminary antibacterial activity of zirconium NPs represented considerable antibacterial potential against Gram-negative bacteria. Overall, the current study demonstrated a novel bio-based approach for preparation of zirconium NPs. Further studies are required to expend this laboratory-based investigation to an industrial scale owing to their superiorities over traditional physicochemical methods such as cost-effectiveness and eco-friendliness.

## Introduction

Nanotechnology is a multidisciplinary field of research covering different technologies like physics, biology, chemistry, engineering, *etc.* in the scale of 1 to 100 nm (1, 2). Nanobiotechnology is an offshoot of nanotechnology dealing with the design, fabrication, and manipulation of materials at nanoscale using biological approach or for the benefit of biological systems (3). Synthesis of nanomaterials using biological methods has attracted significant interest in the field of nanobiotechnology using different plants (4-10), algae (11, 12), fungi (13-15), bacteria (16), yeasts (17), marine organisms (18), and natural biomolecules (19, 20). Nanomaterials in general and metallic NPs in particular possess unique physical, chemical and biological properties that are specific and are not seen in their bulk materials (14, 15). This unique physicochemical properties along with other specific features of metallic NPs like small size, high surface to volume ratio, surface charge, surface chemistry provide a significant chance to not only benefit from intrinsic pharmaceutical potential of these NPs, but also to design multifunctional NPs by conjugating these NPs with therapeutic and diagnostic agents for theranostic purposes as well as targeted drug delivery systems (21, 22). For example, modified selenium NPs were used successfully not only for dual-delivery of siRNA and cisplatin to tumor in mice model, but also for targeting drug delivery of doxorubicin toward breast cancer cells (23, 24). Biological-mediated synthesis of NPs has superiority over traditional physical and chemical methods due to its simplicity, biocompatibility, cost-effectiveness, and eco-friendliness (14, 15, 25). The use of hazard reagents through chemical approach and also need for high amount of energy and cost through physical approach for preparation of NPs are some drawbacks that encourage the researchers to adopt a bio-approach for synthesis of NPs (14, 15). 

Zirconium is a transition metal and is almost similar to titanium from the view of considerable resistance to corrosion (26). Zirconium is commonly used through implant biomaterials and dental crown (26, 27). Previous studies reported the role of zirconium NPs as biosensors, anticancer, antimicrobial, antioxidant, and implants (26, 28). Additionally, zirconium NPs were successfully applied as drug delivery carriers for several drugs such as penicillin, alendronate, itraconazole, *etc.* (29). In a study, zirconium NPs in the range of 15-21 nm with spherical morphology exhibited strong antibacterial activity against *Pseudomonas aeruginosa* using disc diffusion method with inhibition zone of 20 mm at the concentration of 100 µg/mL (26). Besides, zirconium NPs were synthesized using marine brown alga *Sargassum wightii* with an average size of 5nm and spherical morphology. 

The algal-mediated synthesized NPs exhibited considerable antibacterial activity against *Bacillus subtilis*, *Escherichia coli*, and *Salmonella typhi* using disc diffusion method (30). Moreover, zirconium NPs were synthesized using the *Eucalyptus globulus* leaf extract in the range of 9-11 nm with spherical morphology. These NPs exhibited strong antioxidant potential as well as significant anticancer activity against human lung carcinoma (A-549) and human colon carcinoma (HCT-116) cells using DPPH and MTT assays, respectively (31). Although biological synthesis includes much more advantages compared to conventional physical and chemical methods, the literature suffers from the lack of enough evidences confirming the success of various biological resources for synthesis of *zirconium NPs*. To the best of our knowledge, rare studies reported synthesis of *zirconium NPs* by exploiting microorganisms. 

The current study was aimed to screen the capability of extracellular synthesis of zirconium NPs through standard species of *Penicillium* family. Furthermore, in previous studies the salts such as potassium hexafluorozirconate (K_2_ZrF_6_) (32), zirconium oxychloride (ZrOCl_2_·8H_2_O) (31), zirconium (IV) oxynitrate (ZrO(NO_3_)_2_) (30), *etc.* were used for green synthesis of zirconium NPs, while in the present investigation the salt zirconium tetrachloride (ZrCl_4_) for the first time was evaluated for bio-mediated fabrication of zirconium NPs. 

## Experimental

*Materials*

Zirconium tetrachloride (ZrCl_4_), sucrose, yeast extract, and other chemical reagents werepurchased from Merck, Germany. Besides, the lyophilized vial of *Penicillium chrysogenum *PTCC 5031, *Penicillium pinophilum* PTCC 5168, *Penicillium aculeatum *PTCC 5167, *Penicillium notatum* PTCC 5074 and *Penicillium purpurogenome* PTCC 5212 were purchased from Iranian Research Organization for Science and Technology, Tehran, Iran. Furthermore,* the bacteria including Staphylococcus aureus *ATCC 25923,* Staphylococcus aureus *ATCC 29213,* Escherichia coli *ATCC 2785 and *Pseudomonas aeruginosa *ATCC 27853 were prepared from Department of Microbiology, Central Research Laboratories, Shahid Beheshti University of Medical Sciences, Tehran, Iran.

*Screening of extracellular synthesis of bio-zirconium nanoparticles*

All standard *Penicillium* species were cultured on modified fluid Czapek Dox broth including 3 g yeast extract and 21 g sucrose in 500 mL distilled water in separated 1000 mL Erlenmeyer flask. The samples were incubated in a rotary shaker incubator (JAL TAJHIZ®, JTSL 40, Iran) at 120 rpm for seven days at 28 °C. Next, to separate the mycelia from the supernatant, the samples were centrifuged at 10,000 rpm by centrifuge (HETTICH®, ROTINA 380R, Germany) for 10 min. Then, 100 mL of zirconium tetrachloride solution in deionized double distilled water with the concentration of 1 and 3 mM was added to 100 mL of each supernatant samples belonging to each *Penicillium* species to reach the final concentrations of 0.5 and 1.5 mM, respectively with different final pH values at 6, 7, 8, 9. Following that, the samples were incubated in shaker incubator at 120 rpm at 28 °C. As a negative control, 100 mL of either a 1 or 3 mM zirconium tetrachloride solution was added to 100 mL of the same modified fluid Czapek Dox broth without fungal culture with the same pH values. Finally, the *zirconium NPs *were separated from the reaction medium by centrifuge (HETTICH®, MIKRO 200, Germany) for 30 min at 15,000 rpm. Then, the synthesized NPs were washed thrice with deionized double distilled water to improve the NPs purification. Eventually, the NPs were dried at 40 °C and stored in vials for further investigations.

*Characterization of bio-zirconium nanoparticles*

The color change from pale yellow to deep yellow was considered as a macroscopic criterion for screening the potentiality of *Penicillium* species for zirconium NPs extracellular synthesis. Moreover, the Tyndall effect was assessed as macroscopic test to confirm the formation of colloidal systems through the samples as complementary macroscopic assay. The average hydrodynamic diameter size, distribution, Polydispersity Index (PdI) and zeta potential of synthesized NPs were determined using Dynamic Light Scattering (DLS) by Zetasizer at 25 °C with a scattering angle of 90° (Malvern instruments, UK). Moreover, the morphology of the NPs was determined by Atomic Force Microscope (AFM) (JPK, NanoWizard II model, Germany) under ambient conditions in non-contact mode by employing silicon nitride tips with varying resonance frequencies at a linear scanning rate of 0.5 Hz and Scanning Electron Microscope (SEM) (MIRA3 model, Czech Republic) operated at 15 kV coupled with Energy Dispersive X-ray (EDX) analysis. The EDX analytical technique was performed to analyze the chemical characterization of NPs. Furthermore, Fourier Transform Infrared (FT-IR) spectrum was conducted over the wavelength range of 400-4000 cm^-1^ to identify the conjugated biomolecules to the surface of NPs by mixing the synthesized NPs with potassium bromide at 1:100 ratio and compressed to a 2-mm semi-transparent disk for 2 min (Agilent, Cary 630 model, US).

*Antibacterial potential of bio-zirconium nanoparticles*

The minimum inhibitory concentrations (MICs) of the synthesized zirconium NPs were evaluated by using broth micro-dilution assay according to the method described elsewhere against Gram-positive bacteria including *Staphylococcus aureus* ATCC 25923, *Staphylococcus aureus* ATCC 29213, and Gram-negative bacteria including Escherichia coli ATCC 27853, and *Pseudomonas aeruginosa *ATCC 27853 (33). Briefly, 100 µL Mueller Hinton Broth medium was added from the first to the twelfth well of 96-well plate. In the next step, 100 µL of colloidal zirconium NPs in the supernatant was added to the first well. Further, 100 µL of the first well was transferred to the second well. This action was continued to the eleventh well. The twelfth well was considered as negative control and thereby no zirconium NPs were added to this well. Then, 10 µL of a 0.5 Mc Farland bacterial suspension was added to all of the wells. Finally, the plate was incubated at incubator for 24h at 37 ºC. The NP concentration pertaining to the well in which no visible growth occurred was considered as MIC (33). The antibacterial activity of zirconium NPs were compared to the supernatant and zirconium salt.

## Results

*Screening of extracellular synthesis of bio-zirconium nanoparticles*

**Figure 1:**
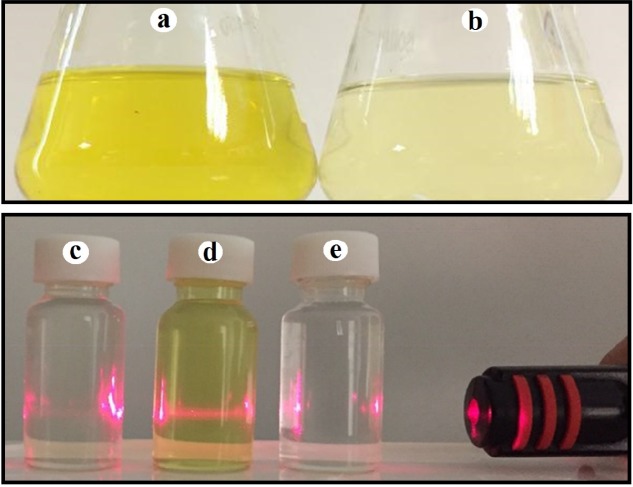
The optical color alteration and Tyndall effect: (a) Colloidal zirconium NPs with deep yellow color; (b) Fungal supernatant with pale yellow color; (c) No visible laser beam path was observed through fungal supernatant; (d) A visible laser beam path was observed through colloidal *zirconium NPs indicating Tyndall effect*; (e) No visible laser beam path was observed through zirconium salt

*According to macroscopic observations, addition of *zirconium tetrachloride solution with the concentrations of 1 and 3 mM to supernatant of *P. chrysogenum *PTCC 5031 and *P. pinophilum* PTCC 5168 with different pH values led to no color change in the solution, while a color change from pale yellow to deep yellow in the solution was observed after seven day incubation in both of final salt concentrations of 0.5 and 1.5 mM and all pH values for *Penicillium aculeatum (P. aculeatum) *PTCC 5167, *Penicillium notatum (P. notatum) *PTCC 5074 and *Penicillium purpurogenome (P. purpurogenome) *PTCC 5212. However, the color change to deep yellow was very significant at the salt concentration of 1.5 mM and pH value of 9 in all three fungi (Figure 1). 

These results suggested the formation of NPs. Moreover, the Tyndall effect confirmed the formation of colloidal systems indicating the formation of *zirconium NPs* using *P. aculeatum *PTCC 5167, *P. notatum* PTCC 5074, and *P. purpurogenome *PTCC 5212 (Figure 1).

*Characterization of bio-zirconium nanoparticles*

**Figure 2:**
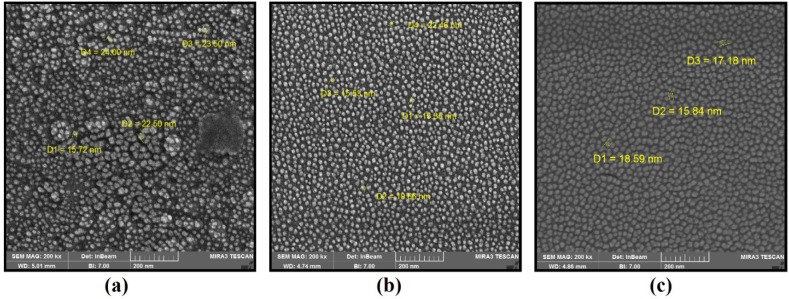
(a) SEM images of *zirconium NPs *synthesized using *P. notatum *PTCC 5074; (b) SEM images of *zirconium NPs *synthesized using *P. aculeatum *PTCC 5167; (c) SEM images of *zirconium NPs *synthesized using *P. purpurogenome *PTCC 5212

**Figure 3:**
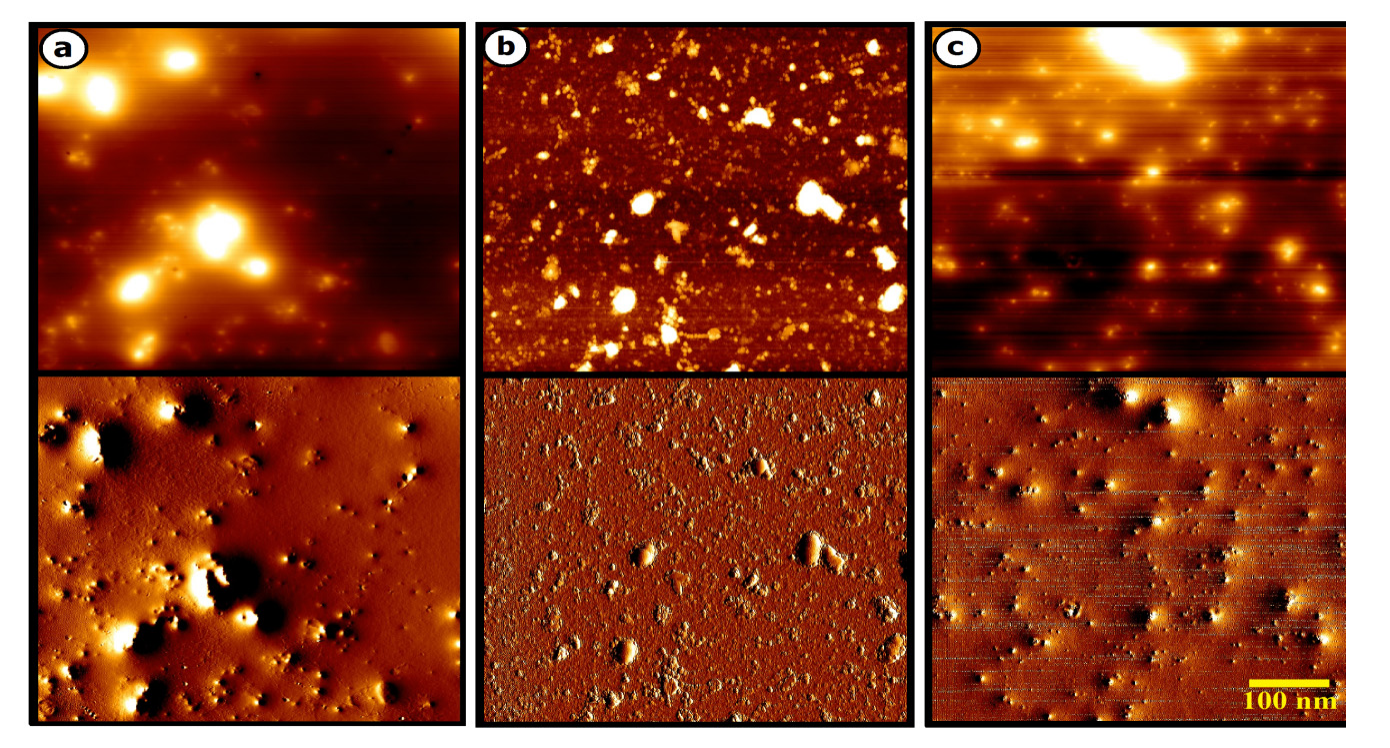
a) AFM images of *zirconium NPs *synthesized using *P. aculeatum *PTCC 5167; (b) AFM images of *zirconium NPs *synthesized using *P. notatum *PTCC 5074; (c) AFM images of *zirconium NPs *synthesized using *P. purpurogenome *PTCC 5212

The characteristic analysis was carried out at the final salt concentration of 1.5 mM and solution pH value of 9 due to considerable color change indicating the high severity of the reaction. The SEM images depicted the spherical morphology of biofabricated NPs with good monodispersity using *P. aculeatum *PTCC 5167, *P. notatum* PTCC 5074, and *P. purpurogenome *PTCC 5212 (Figure 2). Besides, the AFM micrographs in accordance with SEM results firmly confirmed the formation of spherical shaped NPs less than 100 nm through all three fungal-mediated prepared NPs (Figure 3).

**Figure 4:**
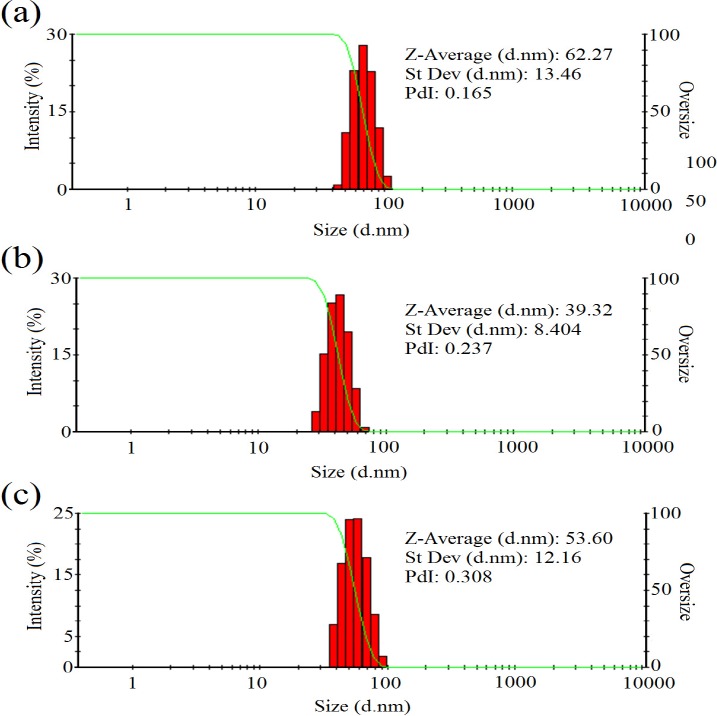
(a) The DLS results of *zirconium NPs *synthesized using *P. notatum *PTCC 5074; (b) The DLS results of *zirconium NPs* synthesized using *P. aculeatum *PTCC 5167; (c) The DLS results of *zirconium NPs *synthesized using *P. purpurogenome *PTCC 5212

**Figure 5:**
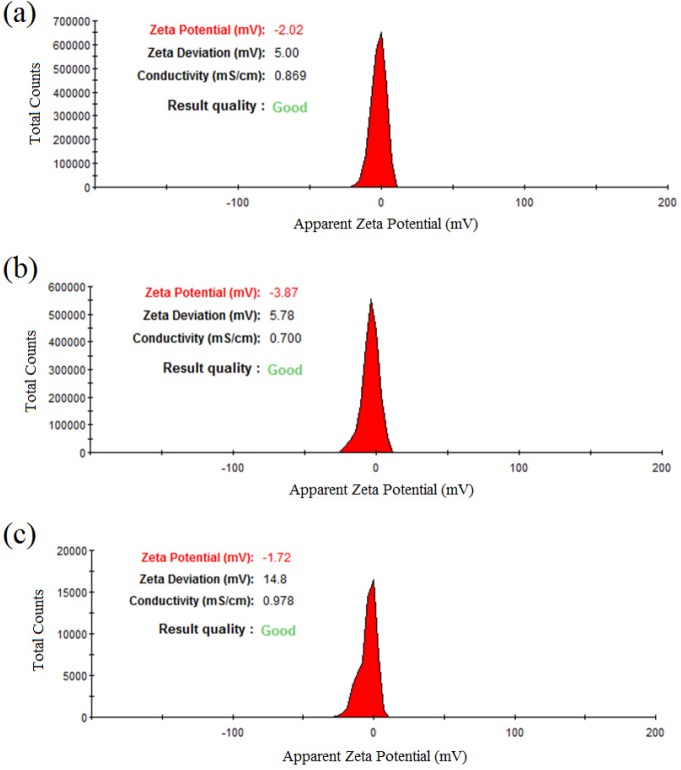
(a) The zeta potential results of *zirconium* *NPs* synthesized using *P.* *notatum* PTCC 5074; (b) The zeta potential results of *zirconium NPs *synthesized using *P. purpurogenome *PTCC 5212; (c) The zeta potential results of *zirconium NPs *synthesized using and *P. aculeatum *PTCC 5167

In addition, Figure 4 represented the DLS results of colloidal zirconium NPs with the final salt concentration of 1.5 mM and pH value of 9 indicating the formation of monodisperse NPs with the hydrodynamic average diameter size of 62.27, 39.32, and 53.60 nm and also PdI of 0.165, 0.237, and 0.308 for NPs synthesized using *P. notatum* PTCC 5074, *P. aculeatum *PTCC 5167 and *P. purpurogenome* PTCC 5212, respectively. Besides, Figure 5 represented the zeta potential results of NPs comprising -2.2 mV, -3.87 mV, and -1.72 mV for colloidal *zirconium NPs* synthesized using *P. notatum* PTCC 5074, *P. purpurogenome *PTCC 5212, and *P. aculeatum *PTCC 5167, respectively.

**Figure 6:**
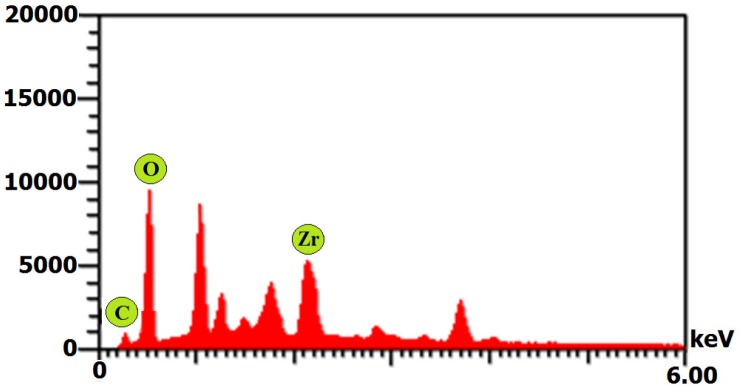
EDX spectrum of zirconium NPs synthesized using *P. purpurogenome *PTCC 5212

**Figure 7:**
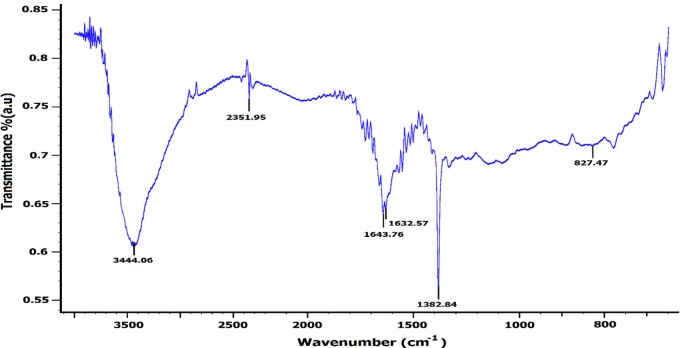
FT-IR spectrum of *zirconium NPs *synthesized using *P. purpurogenome *PTCC 5212

Moreover, the EDX displayed an absorption peak around 2.2 keV indicating the presence of zirconium element in the composition of the synthesized NPs (Figure 6). Furthermore, the FT-IR spectrum of NPs exhibited absorption peaks located at about 3444.06, 2351.95, 1643.76, 1632.57, 1382.84, and 827.47 cm^-1^ in the region 450-4000 cm^-1^ (Figure 7). 

The peaks at 3444.06 and 1632.57 cm^-1^ were assigned to O-H and C–C stretching, respectively (14). The peak appearing at 1643.76 cm^-1^ was assigned to the -OH bending mode or C=O stretching vibration of carbonyl and carboxylic group of amide I (34). The band at 1382.84 cm^-1^ corresponds to the N–H bending of primary amides due to carbonyl stretch (14). The peak at 827.47 cm^-1^ can be attributed to plane bending vibration of N–H groups in the proteins (34).

*Antibacterial potential of bio-zirconium nanoparticles*

The results demonstrated that colloidal *zirconium NPs* in the supernatant that were prepared using *P. purpurogenome* PTCC 5212, were effective against Gram-negative bacteria *with the MIC of 0.75 mM and 0.375 mM* for *E. coli ATCC 27853, and P. aeruginosa ATCC 27853*, respectively; however, the colloidal *zirconium NPs* were not effective in Gram-positive *Staphylococcus aureus (S. aureus) *and no MIC was found at the maximum concentration of 1.5 mM. Notably, the supernatant and also the zirconium salt solution at the maximum concentration of 1.5 mM did not exhibit MIC against both Gram-negative and Gram-positive bacteria.

## Discussion

*Extracellular synthesis of bio-zirconium nanoparticles*

This study demonstrated that from five studied *Penicillium* species, two species were not able to biofabricate the *zirconium NPs* during the above mentioned protocol, while three species synthesized *zirconium NPs*. The color change from pale yellow to deep yellow confirmed the NPs formation. The control without zirconium tetrachloride ions depicted no change in color under the same conditions. The color change is a rapid test that suggest the formation of metallic NPs. For example, the formation of silver, gold, selenium, and tellurium NPs are confirmed when the color alteration to brown, purple, red-orange, and gray black are observed, respectively (14). This color change is attributed to a phenomenon that is named SPR (14). The ratio of surface to the volume is significantly high in NPs and for the case of metal NPs lead to an electron-rich metal surface (22). Hence, in a colloidal system, a large number of free electrons oscillate with specific energy. At this specific energy, any wavelength of light that are absorbed causes an alteration in color (14). Another simple and rapid test to confirm a colloidal system is Tyndall effect which can be described as light scattering by the particles in a colloidal system when a laser beam pass through it (14). In the current study, both of macroscopic observations comprising color change and Tyndall effect corroborated the formation of NPs.

*Characterization of bio-zirconium nanoparticles*

The SEM and AFM images confirmed the formation of well-distributed spherical shaped NPs. Besides, the DLS analysis represented the formation of monodispersed NPs with low PdI values. The range of PdI as distribution index is from 0.01 to 1. The PdI values over 0.7 depict polydispersed NPs (14). Hence, the PdI values of synthesized zirconium NPs using all three Penicillium species were less than 0.7 indicating the formation of monodispersed NPs. The existence of zirconium and oxygen elements were also approved using the EDX analysis indicating the formation of zirconium oxide NPs. Furthermore, the FT-IR analysis revealed the presence of functional groups on the surface of NPs. These functional groups belong to the Penicillium extracellular secreted biomolecules that act as reducing agents to convert the zirconium ions to their nano-forms. Moreover, these biomolecules play a role as stabilizing agents (14). Additionally, these attached biomolecules may interfere with the biological activity of NPs (25). It has been shown that the stability of colloidal NP systems obtained though biological methods is governed by a combination of electrostatic and steric interactions. The ions provide electrostatic stability and the conjugated biomolecules to the surface of NPs provide steric stability. In chemical approach for synthesis of NPs, the stabilizers should be added externally, while in biological approach no external stabilizers are required to be added to the system, because these biomolecules provide steric stability (14).

*Antibacterial potential of bio-zirconium nanoparticles*

The colloidal synthesized NPs in the supernatant showed considerable antibacterial activity against Gram-negative bacteria. Our findings are in good agreement with the previous reports (26, 30). In a study, the biologically synthesized zirconium NPs exhibited significant antibacterial activity against *Bacillus subtilis*, *Escherichia coli*, *Salmonella typhi* using agar well diffusion method (28). This bactericidal activity may be attributed to the oxidation of bacteria by NPs owing to the electromagnetic attractions between the positive charge of metal oxide NPs and the negative charge of microorganisms (26). Although this preliminary study confirmed the antibacterial potential of zirconium nanoparticles, further studies are required to explore the efficacy of these NPs against clinically isolated pathogenic bacteria as well as investigating their antibacterial molecular mechanisms.

## Conclusion

Green synthesis of NPs has attracted significant attention in the recent years due to the superiorities over traditional physical and/or chemical methods. The current study for the first time showed a simple, fast, and cost-effective method for synthesis of zirconium NPs using the supernatant of *P. aculeatum *PTCC 5167, *P. notatum* PTCC 5074, and *P. purpurogenome *PTCC 5212. Moreover, it was discussed that the secreted biomolecules form fungi play a role as reducing and stabilizing agents. The zirconium NPs were formed uniform and well-distributed with the spherical morphology below 100 nm. Although the eco-friendly method for preparation of uniform NPs is still at laboratory setting, it may be developed as a safe alternative method for large scale production of NPs in the near future. Furthermore, the biogenic zirconium NPs exhibited significant antibacterial activities and may be considered as an antibacterial agent in the future.
